# Multilevel influences on family caregiver burden in diabetic foot ulcer care: a qualitative study

**DOI:** 10.1186/s12912-026-04577-0

**Published:** 2026-03-30

**Authors:** Yue Li, Huiren Zhuang, Yili Gao, Qiaoyun Yan, Lin Du, Yanni Hu, Zhifang Zhao, Ruohan Lin, Caiya Liang, Yina Xiao, Zheng Guan, Haiping Yu

**Affiliations:** 1 Shanghai East Hospital, School of Medicine, Tongji University, Shanghai, China; 2 Li Qi Dressing Room, Shanghai Second People’s Hospital, Shanghai, China; 3 Wound Clinic, Shanghai Yangsi Hospital, Shanghai, China; 4 Department of Endocrinology, Hainan General Hospital, Hainan, China; 5 Department of Nursing, Jinyang Community Health Service Center, Pudong New Area, Shanghai, China; 6 School of Medicine, Tongji University, Shanghai, China; 7 Department of Nursing, Pudong Gongli Hospital, Shanghai University of Medicine & Health Sciences, Shanghai, China

**Keywords:** Care burden, Caregivers, family, Diabetic foot ulcer, qualitative research, social determinants of health

## Abstract

**Aim:**

To explore multilevel determinants of caregiver burden in the care of people with diabetic foot ulcers in China and guide culturally relevant support in non-Western settings.

**Methods:**

Sixteen family caregivers of patients with diabetic foot ulcers were purposively recruited from four medical institutions in Shanghai. Data were collected through semi-structured interviews and analyzed using hybrid thematic analysis framed by the Social Ecological Model. Reporting followed the Consolidated Criteria for Reporting Qualitative Research (COREQ).

**Results:**

Four themes and twelve subthemes were identified: (1) Individual Capacities and Internal Struggles; (2) Interpersonal Strain and Social Support Challenges; (3) Health System Deficiencies and Access Barriers; (4) Policy Limitations and Cultural Burden .

**Conclusions:**

Caregiver burden in care of patients with diabetic foot ulcers is influenced by personal, family, institutional, and cultural factors. Context-sensitive approaches that integrate system reforms, community support, and cultural norms are needed, with insights from China informing care strategies in other non-Western settings.

**Clinical trial number:**

Not applicable.

**Supplementary Information:**

The online version contains supplementary material available at 10.1186/s12912-026-04577-0.

## Background

Diabetic foot ulcers (DFUs) are among the most severe and costly complications of diabetes, affecting up to 25% of diabetic patients during their lifetime and contributing significantly to morbidity, disability, and premature mortality [1, 2]. They are a leading cause of non-traumatic amputations and recurrent hospitalizations, with poor wound healing and frequent infections posing ongoing clinical challenges [3, 4]. Beyond these clinical consequences, DFUs also place a substantial emotional and psychological burden on family caregivers, who often assume primary responsibility for daily wound management and care coordination [5, 6]. At the household and system levels, long-term DFU management generates considerable economic strain, with average treatment costs exceeding USD 10,000 per hospitalization [7]. Despite these multilevel burdens, much of the existing literature continues to prioritize biomedical management, with comparatively limited attention to the experiences and support needs of family caregivers.

Family caregivers—often spouses, adult children, or other relatives—play a pivotal role in the home-based management of DFU, especially following hospital discharge. They are responsible for a wide range of tasks, including wound care, glucose monitoring, medication management, mobility assistance, and coordination with healthcare providers [8, 9]. This support not only bridges service gaps in formal healthcare systems but also contributes to improved patient outcomes, such as enhanced wound healing, reduced complications, and lower readmission rates [10, 11]. However, caregiving for DFU is complex and time-intensive, requiring skills and knowledge that many informal caregivers lack. Without adequate training or formal support, caregivers may experience physical exhaustion, psychological stress, financial hardship, and social isolation [12, 13]. Despite these challenges, research on DFU care remains limited. Most studies have been conducted in high-income countries and mainly focus on diseases such as dementia or cancer. As a result, care-related issues associated with DFU remain underexplored.

Internationally, across the broader healthcare context, multiple caregiver-oriented interventions—including respite care, telemedicine, structured training, and support programs—have been widely applied to patients with long-term care needs, with substantial evidence demonstrating their effectiveness in reducing caregiver burden and improving both caregiver well-being and care quality [14, 15]. Yet these measures are often embedded within well-established healthcare infrastructures and cultural contexts that may not translate effectively to other settings [8]. In China, formal caregiver support systems remain in their early stages, with pilot programs for long-term care insurance, home-based medical services in select cities, and limited caregiver training for chronic wound care [16, 17]. The majority of long-term care for chronically ill or bedridden patients is still provided by family members, due to low utilization of institutional or third-party services [4]. Moreover, within the Confucian cultural framework, strong filial piety norms dictate that caring for aging or ill parents is not only a moral duty but also a measure of personal virtue; failure to provide such care may be socially condemned as unfilial [18]. While these values can foster family commitment, they may also intensify caregiver burden, as individuals face heightened social pressure, internalized guilt, and limited acceptance of external help. This unique cultural dynamic further complicates caregiving experiences in Chinese and other East Asian contexts.

Given the heavy clinical, economic, and psychosocial burdens associated with DFU, and the pivotal role family caregivers play in patient outcomes, there is an urgent need for research that systematically examines caregiving experiences within specific cultural and systemic contexts. Existing studies rarely incorporate robust theoretical frameworks capable of capturing the interplay between individual, interpersonal, institutional, and sociocultural influences. The Social Ecological Model (SEM), originally conceptualized within ecological systems theory and widely applied in public health research [19, 20], offers a comprehensive framework for multilevel analysis of caregiving burden. Applying SEM in the Chinese context is particularly valuable, as it accommodates the intersection of demographic aging, fragmented healthcare support systems, and deeply rooted cultural expectations. Moreover, these cultural dynamics are not confined to China alone but extend across the broader Sinophone and Confucian-influenced world, including many developing countries in East and Southeast Asia. In such contexts, strong Confucian norms of filial piety (xiao) reinforce family-based caregiving as both a moral duty and a socially prescribed expectation, while underdeveloped formal care infrastructures limit available alternatives [21, 22]. This cultural-structural interplay distinguishes caregiving experiences in Confucian societies from those in Western countries, where institutional support is more accessible and filial obligations are less central. Recognizing these shared cultural underpinnings underscores the importance of investigating DFU caregiving in Chinese-speaking and Confucian cultural contexts more broadly, generating insights that are both contextually grounded and regionally transferable [18, 23]. This study therefore seeks to address the critical gap in understanding DFU family caregiving in non-Western settings, generating culturally grounded and system-level insights that can inform targeted interventions, enhance caregiver support, and ultimately improve patient care outcomes.

## Materials and methods

### Aim

This study explores the caregiving challenges and emotional impact experienced by family members caring for patients with DFUs in China from a multilevel perspective, in order to inform culturally relevant support in non-Western contexts.

### Design

This study employed a descriptive qualitative design, guided by the Consolidated Criteria for Reporting Qualitative Research (COREQ) checklist [24], to explore family caregivers’ experiences in caring for patients with DFUs through semi-structured interviews. The interview guide was developed based on findings from existing systematic reviews on family caregiving in chronic illness contexts [13, 25], and subsequently reviewed by two clinical experts in diabetic foot ulcer care. Their feedback contributed to refining the question phrasing and expanding the thematic scope to ensure clinical relevance. To strengthen conceptual continuity, key findings from the guiding reviews were first mapped to the core levels of the Social Ecological Model (individual, interpersonal, institutional, and sociocultural/policy). This mapping informed the organization of interview prompts across levels, while allowing participants to introduce experiences beyond the predefined domains. The interview guide was pilot tested with two family caregivers to assess clarity, flow, and relevance. Minor adaptations were made to simplify wording and improve the sequencing of questions, while the core thematic structure remained unchanged throughout the interview process.

The research team included two nurse researchers with doctoral-level training and more than 10 years of experience in qualitative health research, and one senior wound care nurse with more than 20 years of clinical experience in diabetic foot ulcer care, who provided methodological oversight and quality assurance throughout the study. Interviews were conducted by trained members of the research team—one female (YL) and one male (ZG)—neither of whom had any prior relationship with the participants. Prior to each interview, participants were informed that the interviewers were independent academic researchers with no role in their clinical care, and that the study aimed to explore family caregiving experiences for research purposes. This was intended to help reduce potential power imbalances and social desirability in participants’ responses. Participants were recruited through purposive sampling from four medical facilities in Shanghai. Each participant engaged in a face-to-face, one-on-one interview, during which they were encouraged to share their caregiving experiences and perceived burden in depth.

### Theoretical framework

This study was underpinned by the Social Ecological Model (SEM), which recognizes that caregiving is influenced by multiple, interrelated levels—individual, interpersonal, institutional, and sociocultural [26, 27]. SEM provided a structured lens to contextualize and interpret how caregiver burden and emotional responses are shaped not only by personal capacity but also by broader social expectations and systemic constraints. To capture both structural context and lived experience, SEM was complemented by a phenomenological approach, allowing deeper interpretation of caregivers’ moral reasoning, emotional struggles, and sense-making. This dual approach supported a nuanced understanding of how personal caregiving experiences are embedded within—and shaped by—wider cultural and institutional systems [28, 29].

### Study setting and recruitment

Sixteen family caregivers of patients with DFUs were recruited through purposive sampling at four medical facilities in Shanghai, including two tertiary hospitals, one secondary hospital and one community healthcare center. Potential participants were initially screened by frontline nurses and physicians in diabetic foot clinics and wards based on the predefined inclusion criteria. Head nurses or designated specialist nurses then served as site liaisons to coordinate the recruitment process. Following this, the research team approached caregivers during outpatient visits or hospital stays, introduced the purpose, procedures, and voluntary nature of the study, and provided detailed information regarding confidentiality and audio-recording. Caregivers who expressed interest were then invited to participate, and interview appointments were scheduled at a convenient time. All interviews were conducted within the recruitment sites (e.g., private consultation rooms or medical offices) to ensure privacy and confidentiality.

Recruitment continued until thematic saturation was achieved, defined as the point at which no new themes or insights emerged from the interviews. All sixteen invited caregivers completed interviews without withdrawal.

### Inclusion and exclusion criteria

Eligible participants were aged 18 or older, able to understand the interview questions and express their experiences clearly, and functionally independent in daily living, with sufficient physical ability to provide basic care to others. Participants were required to be the primary unpaid family caregivers of patients with DFUs. Family caregivers who received government subsidies or caregiving allowances (e.g., carer’s payments) were considered eligible, provided that they were not formally employed as paid caregivers.

Individuals who were themselves dependent on others for daily living, had severe physical illnesses (e.g., serious cardiovascular, pulmonary, or renal diseases), had diagnosed psychiatric disorders, or were professionally employed as paid caregivers were excluded [30]. The inclusion and exclusion criteria are detailed in Table 1.

**Table 1:** Inclusion and exclusion criteria

Inclusion Criteria	Exclusion Criteria
Being the primary family caregiver for a person with DFUs	Professionally employed paid caregivers
Age > 18 years	Age < 18 years
Functionally independent in daily living and physically able to provide basic care	Dependent on others for daily living with severe physical illnesses
Clear consciousness and ability to understand interview questions	Diagnosed mental disorders
Ability to express personal experiences clearly	Difficulty understanding the local dialect

### Data collection

Face-to-face semi-structured interviews were conducted at four different medical facilities in Shanghai between March and June 2024. At the time of data collection, 11 caregivers were providing home-based care for patients attending outpatient follow-up visits, whereas five caregivers were supporting patients who were still hospitalized on inpatient wards. Accordingly, interviews were conducted in private consultation rooms or medical offices. The interviewer employed a study-specific interview guide and conducted the interviews in Mandarin. To establish rapport with the participants, the interviewer commenced the interview with a brief, engaging discourse that fostered a sense of ease and respect. To connect the inquiries to the study’s objective, participants were invited to describe their motivations for caregiving in detail. The interviews were audio-recorded for subsequent transcription, and the semi-structured interviews lasted approximately 30 min on average. The interview guide is provided in Table 2.

**Table 2:** Interview outline (thematic guide)

λ Can you describe how you assist the patient with DFUs in daily life?
λ How do you feel as a family caregiver? Have you encountered any difficulties in the process of caring? If so, what are they and how did you overcome them?
λ Do you receive support from family, friends, or the community in caring for the patient? What are their attitudes? Did the supports meet your expectations?
λ Have your community and community health workers offered you any help or guidance on care?
λ Have medical staff given you adequate knowledge of home care? Do you think it is useful?
λ What do you think about the Social and Medical Policy? Is it helpful?
λ What’s your motivation for being a family caregiver?

### Data analysis

After the interviews, two researchers independently transcribed the audio recordings verbatim in Mandarin Chinese within 24 h. All coding and analysis were conducted on the original Chinese transcripts to ensure semantic accuracy and preserve cultural meaning, and English translation was limited to the reporting stage for the presentation of representative quotations. Qualitative data were managed and organized using NVivo 12 Plus (QSR International Pty Ltd., 2021) [31] to support systematic coding, retrieval, and comparison of textual data. The analytic process itself remained researcher-driven, with all codes and themes generated through manual interpretive work by the research team rather than being generated through automated software functions The transcripts were coded line by line using a hybrid inductive–deductive approach, in which inductive coding was first applied to generate initial codes grounded in participants’ accounts. These codes were continuously compared and refined through regular consensus meetings to ensure analytic rigour and consistency. At the thematic level, deductive comparison was then used to relate emergent themes to the SEM framework for interpretation and presentation. Qualitative data were analyzed using a hybrid thematic-content analysis approach, with Colaizzi’s method [32] guiding the initial extraction of significant statements and meaning formulation. The resulting codes were grouped into themes and subthemes through inductive analysis; these themes were then iteratively examined in relation to the SEM levels to situate caregivers’ experiences across individual, interpersonal, institutional, and sociocultural/policy domains. SEM was used as an organizing lens for interpretation and presentation, rather than a prescriptive template for code generation. This combined approach preserved the authenticity of participants’ narratives while providing conceptual clarity across individual, interpersonal, institutional, and sociocultural levels.

The analytic process followed seven iterative steps, adapted from Colaizzi’s phenomenological method: (1) Audio recordings were transcribed verbatim. (2) Two researchers reviewed the transcripts repeatedly and highlighted significant statements. (3) Meanings were extracted from significant terms. Significant expressions were determined based on those commonly utilized in discourse with the family caregivers. (4) Themes and sub-themes were established by categorizing the meanings. For example, Individual Capacities and Internal Struggles towards caregiving tasks: (“Physical and Emotional Strain”, “Low Self-efficacy and Uneven Coping Abilities”, “Moral Responsibility and Learning Drive”) were categorized under the main theme of Individual Capacities and Internal Struggles. (5) Generated themes and subthemes were checked against the transcripts to assess their alignment with caregivers’ accounts. (6) The fundamental framework for caregivers’ experiences was constructed. (7) Participants did not review transcripts or themes; instead, a verbal summary of their interviews was provided. Accuracy was ensured through repeated transcript checks and collaborative coding.

Themes and subthemes were reviewed through regular team discussions, and differences in interpretation were resolved through consensus. Where necessary, a third team member was consulted to support resolution of disagreements. Codes were refined by consolidating overlapping categories and clarifying distinctions between participants’ descriptive statements and higher-level interpretive categories.

When codes reflected multiple conceptual domains, theme assignment was guided by the primary analytic focus of each excerpt rather than by surface content alone. For example, excerpts reflecting both self-efficacy and moral responsibility were classified based on whether the participant’s narrative primarily emphasized internal coping capacity or value-driven caregiving commitment at the thematic level. Subsequently, excerpts were further classified according to whether the participant’s account centered on internal appraisal and perceived capacity (individual level), relational dynamics and interactional patterns (interpersonal level), or health system, policy, and cultural conditions shaping caregiving roles (institutional and sociocultural/policy levels). Alternative interpretations were discussed during team-based analysis, and final placement was reached through consensus to ensure conceptual coherence.

The themes were substantiated using representative quotations [33]. All translated quotations were reviewed by bilingual researchers to ensure semantic and conceptual consistency between the Chinese and English versions.

### Rigour

This section outlines how key COREQ domains were addressed to enhance methodological transparency and trustworthiness. Research reflexivity was maintained across the stages of study design, data collection, and analysis, with interviewers reflecting on their professional assumptions regarding DFU caregiving prior to and during data collection. Details of COREQ compliance are provided in Supplementary Table S1 (Appendix).

### Ethical considerations

The participants were approached before the scheduled interviews and afforded ample time to consider their participation. This study was conducted in accordance with the principles of the Declaration of Helsinki and was approved by the Medical Ethics Committee of Shanghai East Hospital of Tongji University, China (Approval Nos.: [2022] Research Preliminary Review No. 208; [2023] Research Preliminary Review No. 062). Verbal informed consent was obtained from all participants prior to each interview and audio-recorded in accordance with the approved ethical protocol.

## Results

### Socio-demographic findings

A total of 16 family caregivers of patients with DFUs participated in the study. They were recruited from outpatient clinics (*n* = 11) and inpatient wards (*n* = 5) of four medical facilities in Shanghai. The caregivers were aged 34–71 years (M = 57.1, SD = 12.7); six were male and 10 were female. In terms of education, one had completed primary education, seven had completed secondary education, four held high school diplomas, and four had a university degree or higher. Thirteen caregivers lived in urban areas, while three resided in rural areas. Eleven were retired, four were employed, and one was unemployed. Regarding their relationship with the patient, nine were spouses, four were children, two were parents, and one was a sibling. Eight caregivers provided care alone, while four shared care with one person, two with two people, one with three people, and one with 11 co-caregivers. Caregivers’ sociodemographic characteristics and the corresponding patients’ demographic and clinical information—including age range, income level, amputation level, and DFU severity (Wagner grade)—are summarised in Table 3.

**Table 3:** Sociodemographic characteristics of each family caregivers and their patients

Participant	Caregiver							Patient				
	Age Group	Education Level	Relationship to Patient	Residence	Work Status	Duration of Care	Co- caregiver	Age Group	Gender	Monthly Income (RMB)	Amputation Level	Wagner Grade
P1	60–69	Secondary Education	Wife	Urban	Retired	3 years	0	70–79	Male	3000- 6000	Toe	Grade 3
P2	60–69	Secondary Education	Father	Urban	Retired	4 years	1	30–39	Male	<3000	None	Grade 3
P3	50–59	High School Diploma	Wife	Urban	Retired	1.5 months	0	60–69	Male	>12,000	None	Grade 2
P4	30–39	Secondary Education	Wife	Urban	Unemployed	4 months	0	50–59	Male	<3000	Toe	Grade 4
P5	60–69	High School Diploma	Wife	Urban	Retired	2 years	0	60–69	Male	6001- 9000	None	Grade 2
P6	40–49	University Degree or above	Son	Urban	Employed	3 years	0	60–69	Male	<3000	Toe	Grade 4
P7	50–59	High School Diploma	Wife	Urban	Retired	4 years	0	60–69	Male	3000- 6000	Partial foot and Toe	Grade 5
P8	60–69	Secondary Education	Wife	Urban	Retired	2 years	0	70–79	Male	3000- 6000	Toe	Grade 4
P9	60–69	Secondary Education	Daughter	Urban	Retired	9 years	1	≥ 80	Female	3000- 6000	Toe	Grade 2
P10	70–79	Secondary Education	Mother	Urban	Retired	15 years	1	40–49	Male	3000- 6000	None	Grade 2
P11	60–69	Secondary Education	Brother	Rural	Retired	2 years	1	70–79	Male	3000- 6000	None	Grade 2
P12	30–39	University Degree or above	Grandson	Rural	Employed	5 years	11	≥ 80	Female	3000- 6000	Toe	Grade 1
P13	60–69	University Degree or above	Wife	Urban	Retired	3 years	2	60–69	Male	>12,000	Toe	Grade 4
P14	50–59	Primary Education	Wife	Rural	Employed	2 months	0	50–59	Male	<3000	Toe	Grade 4
P15	30–39	University Degree or above	Grandson	Urban	Employed	1.5 months	3	70–79	Male	3000- 6000	None	Grade 1
P16	70–79	High School Diploma	Husband	Urban	Retired	5 years	2	60–69	Female	>12,000	Toe	Grade 1

### Themes

The codes derived were organized into four interlinked main themes based on the SEM framework. Themes and sub-themes identified in this study are presented in Table 4. The four major themes were: (1) Individual Capacities and Internal Struggles; (2) Interpersonal Strain and Social Support Challenges; (3) Health System Deficiencies and Access Barriers; (4) Policy Limitations and Cultural Burden.

**Table 4:** Themes, sub-themes and description from interviewees

Theme	Sub-Themes	Description from interviewees
**Individual Capacities and Internal Struggles**	**Physical and Emotional Strain** **(** ***n*** **= 16)**	λ **“**Taking care of him is a challenge for me. My physical condition is not well. **I’ve had rheumatoid arthritis for more than 10 years and my spine and limbs always hurt**…. Although I can still help him do chores right now, how about in the future…. I cannot imagine.**”** (Participant 10)
		λ **“**My physical strength is not enough. **Because of getting old and lack of rest**,** I feel my physical energy cannot keep up** when I take care of my mom.**”** (Participant 9)
		λ **“The fear of the unknown made me feel desperate**… I did not know what would be in the future and whether I could handle all of these difficult situations by myself.**”** (Participant 7)
		λ **“I felt anxious and self-blame** when his blood sugar was not well controlled. **I thought it was my fault** for not doing enough to take care of him. When his wound condition became worse, **I had a strong sense of guilt**. All these negative emotions can only be digested slowly by myself and I don’t want him to know this.**”** (Participant 4)
		λ **“**Sometimes, **I really want to escape** from the situation, but I cannot!**”** (Participant 2)
	**Low Self-efficacy and Uneven Coping Abilities** **(** ***n*** **= 11)**	λ **“**Diet is difficult to control and his blood sugar always fluctuates. **I didn’t know how to handle** the whole situation. **I had the thought to give up for one thousand times**, but I can’t!**”** (Participant 4)
		λ **“**To be his mom and a caregiver, **I am a loser**. I said it was good for his health to go to bed early, but he still kept sleeping late. I said doctor’s recommendation for his diet was nutrition balanced foods with calorie controlled, but he didn’t listen. Now, his condition is getting worse.**”** (Participant 10)
		λ **“**I help him deal with the wound at home following the requirements of the doctor. **I even learned some simple techniques to deal with the callus from social media**. With the technique, I could pay special attention to his wound.**”** (Participant 6)
		λ **“**I have seen instructions about DF diet in some media. And then **I learned to recognize** which food belongs to low GI Foods and **knew how to match** nutritional food scientifically. For myself, **my diet became much healthier too**.**”** (Participant 5)
		λ **“**You see, I am 62 years old and only have a junior high school education background. **It is really difficult for me to search information on the Internet like a young person.”** (Participant 11)
	**Moral Responsibility and Learning Drive** **(** ***n*** **= 13)**	λ **“**She is our only grandparent right now. **All the family members consider that we have the responsibility to take care of her**. We do everything for her including helping her take medicine and inject insulin.**”** (Participant 12)
		λ **“**I thought, **as his son**, **it was my duty to support him and take good care of him**. In order to let him regain confidence for recovery, I worked hard to learn the knowledge of diabetic foot through different ways, and also helped him deal with the wound at home with the instructions of our doctor.**”** (Participant 6)
		λ **“He’s one of the dearest members in my family. As long as he is alive**,** my family is still unbroken**. Can you understand me? Maybe some people are not. They don’t want to be involved, they want to be free. But I am not like that, I still think of him…” (Participant 13)
**Interpersonal Strain and Social Support Challenges**	**Family Relationship Quality and Coping Styles** **(** ***n*** **= 8)**	λ **“**When encountering problems, **our family members would get together to discuss solutions and make a final decision**. If the final result was not as good as expected, **no one was blamed**.**”** (Participant 12)
		λ **“My son and daughter-in-law are a great support to us**. Every time my wife is in a bad mood because of the feet, they will do something to make her happy. When I feel tired, they will find ways to relieve my stress, such as hiring a paid caregiver or they will take the responsibility to take care of her.**”** (Participant 16)
		λ **“My brother (the patient) and his wife divorced long time ago**. **His son didn’t contact him for a long time**. The only person he can rely on is me or my sister. **My wife always gets pissed off**, when she knew that I will go to my brother’s place to take care of him for a while. **I have no choice but to send him to the nursing home**.**”** (Participant 11)
	**Financial Strain and Resource Drain** **(** ***n*** **= 7)**	λ **“**Last time, when we went to the hospital for follow up, our doctor said, if we want to keep the leg without amputation, **an interventional operation was needed which may cost hundreds of thousands** (not sure about the exact price). And **a special shoe was also needed which was not cheap**… Furthermore, his eyes and kidneys also have problems. **All the difficulties are about money**…**”** (Participant 10)
		λ **“The (off-loading) shoes** recommended by doctors for DFU self-care **were so expensive**, like several thousand RMB. I think it cannot be accepted by most ordinary people.” (Participant 4)
		λ **“**Taking care of him means **I need to reduce my working hours.** If he stays in my hospital, I can take care of him while I work, but the hospital is also very expensive, and **I have to contribute a large part of my salary to the hospital**.**”** (Participant 14)
		λ **“Frequent requests for personal leave also brought a lot of pressure to my career**. Because my workload became less, naturally my colleagues bear more. However, in fact, they have no obligation to cover my part of work.**”** (Participant 6)
	**Social Withdrawal and Lack of Community Support** **(** ***n*** **= 11)**	λ **“**My friends don’t hang out with me as often as before because they know I can’t go out. Even if I go out with them, **I couldn’t stay outside for long**. I began to recognize that **I had no common topics with them**.**”** (Participant 1)
		λ **“**Since he was sick, **I’m not in the mood to go out with anyone**. I think about him all the time.**”** (Participant 13)
		λ **“**When my brother lived alone at home, I paid meal delivery fee monthly to his neighborhood committee. At meal time, the members of this committee sent his meals by hanging the delivery food on the doorknob. Recently, I found something was wrong with him, because I got feedback from his neighborhood committee that he didn’t open the door to pick up food for a whole day. **Despite that**,** he couldn’t get more concern from other institutions**.**”** (Participant 11)
		λ **“The neighborhood committee won’t come up to help an outsider who just rents a room in the community**. Now I let him live in my work place so that I can take care of him while I work.**”** (Participant 14)
**Health System Deficiencies and Access Barriers**	**Inadequate Communication with Healthcare Providers** **(** ***n*** **= 5)**	λ **“**Some doctors just say that you can’t eat this and that, but do not tell what to eat and how to eat. Once the blood sugar was abnormal, they were convinced that you were not following their advice. **This kind of communication made me so frustrated**.**”** (Participant 5)
		λ **“**We don’t know much about the disease. Although the nurse already gave us some information about wound dressing, we still didn’t know how to change the dressing at home **with the limited consultation time.”** (Participant 3)
	**Limited Access to DFU-Specific Caregiver Education** **(** ***n*** **= 6)**	λ **“No one told us that diabetes would have such a serious complication**. We didn’t pay attention to regular health checks, otherwise he could not be amputated.**”** (Participant 4)
		λ **“**Honestly, **we are not very clear about home care instructions of diabetic foot**. In fact, we have seen the health education column on the wall of the hospital, but the **information content is too little and general**, which is difficult to apply to unique patients.**”** (Participant 15)
	**Primary Care and Home Care Infrastructure Shortcomings** **(** ***n*** **= 7)**	λ **“**He is completely paralyzed in bed. If he is discharged from the hospital, **every time we go to the hospital for dressing change will be a big project for our family**.**”** (Participant 13)
		λ **“**The healthcare providers in my grandma’s community healthcare center reviewed her health conditions by making phone calls once in a while, asking a few simple questions such as “How are you doing recently?” and “Have you checked your blood glucose and what is it?” Besides this, there was nothing else, no home visit, no treatment… **There is no real on-the-ground service at all**.**”** (Participant 12)
		λ **“Community healthcare center can’t handle wounds like this**. So we have to come to a tertiary hospital three times a week for dressing changes.**”** (Participant 7)
**Policy Limitations and Cultural Burden**	**Social Insurance Inequity and Policy Fragmentation** **(** ***n*** **= 5)**	λ **“The reimbursement rate** of medical insurance is **50% for us in grade A tertiary hospitals**. **Second grade hospitals**, medical **insurance reimbursement is 70%**. So, we dare not stay too long.**”** (Participant 14)
		λ **“Public hospitals impose strict length-of-stay restrictions**, forcing patients to go through discharge-and-readmission process every 15 to 30 days.**”** (Participant 13)
	**Family Structure Challenges under the One-Child Policy** **(** ***n*** **= 9)**	λ **“I am the only child**. My mom died a few years ago. At present, my father lives alone, but we live so close that I can go there every day… **There is definitely pressure on my shoulder**. I have no brothers or sisters. I often feel limited on time since all my father’s home care relies on me… My wife nearly undertakes all the tasks of childcare. I feel so guilty for them…**”** (Participant 6)
		λ **“**Although taking care of him drained my energy, I still don’t want to trouble my son. **He is my only child. He was already so exhausted for his own life.”** (Participant 7)
		λ **“He’s unmarried**,** has no children and no job**. I am getting old. Taking care of him already exceeded my ability. I can’t imagine **if I were to die**,** who would take care of him**.**”** (Participant 2)
	**Gendered Roles and Filial Piety Pressures** **(** ***n*** **= 11)**	λ **“Elders think that men work outside and women need to take care of the household**. So, after marriage, I quit my job to care for the whole family. Right now, he can’t go work anymore. We have no financial resources.**”** (Participant 4)
		λ **“**Children have to work, **as a mother** who wants to put pressure on them.**”** (Participant 1)
		λ **“I was raised by my grandfather since childhood**. Now he has **become a left-behind elder**, and as he falls ill I find myself unable to care for him properly, **which makes me feel very guilty**.**”** (Participant 15)

Notes: (1) n refers to the number of participants who explicitly reported experiences related to each sub-theme. A single participant may contribute to multiple sub-themes. (2) Theme assignment reflects the primary analytic focus of each excerpt (individual, interpersonal, or sociocultural/policy level), as described in the Data Analysis section, rather than surface content alone

#### Individual capacities and internal struggles

##### Physical and emotional strain

Many caregivers reported experiencing deteriorating physical health and persistent emotional distress during the long-term caregiving process. Common symptoms included chronic fatigue, musculoskeletal pain, sleep disruption, and psychological strain, including feelings of stress, anxiety, and low mood. These burdens often accumulated over time, intensifying the caregivers’ subjective sense of strain and diminishing their caregiving capacity.

##### Low self-efficacy and uneven coping abilities

Caregivers varied in their perceived self-efficacy, which was closely tied to their ability to acquire and apply caregiving knowledge. Some participants actively sought out information and developed practical skills through self-education or informal training, enhancing their confidence in managing wound care. Others, particularly older or less literate caregivers, expressed helplessness due to difficulty accessing or understanding information. These disparities shaped both emotional burden and the perceived effectiveness of caregiving performance.

##### Moral responsibility and learning drive

Beyond practical tasks, many caregivers framed their commitment as a moral obligation rooted in traditional values such as filial piety or spousal responsibility. This sense of duty often drove sustained caregiving efforts even in the absence of institutional support. For some, learning about DFU care became a way of fulfilling this moral role and constructing personal meaning under challenging conditions.

#### Interpersonal strain and social support challenges

##### Family relationship quality and coping styles

Our findings suggest that the emotional experience and perceived burden of DFU caregivers are closely associated with the quality of family relationships. Caregivers embedded in families with harmonious dynamics, effective communication, and shared decision-making reported significantly lighter burdens and higher resilience. Families demonstrating strong internal coping strategies—such as joint problem-solving and emotional reciprocity—helped buffer the psychological impact of caregiving, even under challenging clinical circumstances. Supportive families offered more than practical help—they gave emotional meaning to the caregiving role. This suggests the interpersonal context serves as both a source of resilience and identity reinforcement.

##### Financial strain and resource drain

Participants frequently described dual pressures: high out-of-pocket treatment costs and income loss due to reduced work hours. These financial strains not only affected household stability but also influenced caregivers’ perceptions of personal adequacy. Many reported sacrificing career advancement or personal well-being to sustain care, which added emotional complexity to their role.

##### Social withdrawal and lack of community support

Many caregivers reported experiencing increasing social isolation as they withdrew from work, friendships, and community engagement in order to focus on caregiving. This withdrawal, while often gradual, led to feelings of loneliness and emotional disconnection. Compounding this issue was the near-total absence of accessible community-based services, such as social work support or primary care outreach. These systemic gaps left caregivers to operate in near-isolation, deepening their emotional and logistical burden. Isolation extended beyond social withdrawal to a sense of disconnection from prior roles and identities, as caregivers described limited community support undermining their autonomy and personal meaning.

#### Health system deficiencies and access barriers

##### Inadequate communication with healthcare providers

Many caregivers reported strained communication with healthcare professionals, primarily due to limited consultation time, fragmented follow-ups, and a perceived lack of empathy or understanding. These barriers hindered caregivers’ ability to clarify care instructions, ask questions, or participate meaningfully in medical decision-making. As a result, inadequate communication often led to confusion and reduced adherence to treatment protocols during home-based care.

##### Limited access to DFU-specific caregiver education

Caregivers commonly reported limited access to clear, targeted education about DFU care. Hospital pamphlets and brief verbal instructions were viewed as overly general or difficult to apply. This lack of structured training contributed to uncertainty in managing wounds, diets, and symptoms at home, deepening caregivers’ feelings of inadequacy and isolation.

##### Primary care and home care infrastructure shortcomings

Structural shortcomings in primary healthcare services further aggravated caregiving difficulties. Participants reported a lack of home visit services, limited options for remote consultations, and a severe shortage of professional wound care personnel in community health centers. These systemic deficiencies created delays in treatment, inadequate support for home-based care, and frequent dissatisfaction with the quality and continuity of medical services. Caregivers described feeling unsupported by the healthcare system, with structural limitations shifting greater responsibility onto families and reimbursement confusion adding emotional and logistical strain. These gaps reflect policy-level disregard for caregiving realities.

#### Policy limitations and cultural burden

##### Social insurance inequity and policy fragmentation

Participants frequently expressed frustration with inconsistencies in medical insurance reimbursement schemes and hospital classification systems. Variations in coverage levels, length-of-stay restrictions, and administrative procedures across institutions added complexity to treatment planning and financial decision-making. Many caregivers reported feeling pressured or constrained when choosing between facilities based not on quality of care, but on what could be reimbursed. These fragmented policies increased psychological stress and financial risk for families managing long-term DFU treatment.

##### Family structure challenges under the one-child policy

The lingering effects of China’s One-Child Policy have significantly impacted the availability of family-based caregiving support. In many cases, a single adult child was solely responsible for caring for aging parents, resulting in emotional and physical overload. This burden was particularly pronounced in urban areas, where caregivers often juggled employment and eldercare simultaneously. In contrast, rural families reported cases where elderly patients with DFUs were left behind with minimal assistance, due to out-migration of adult children for work. Both situations reflect a systemic erosion of multi-generational caregiving capacity. Solo caregiving under demographic pressure evoked deep anxiety about the future. These accounts illustrate how past policy decisions continue to shape private caregiving burdens.

##### Gendered roles and filial piety pressures

Traditional cultural ideals—especially those emphasizing maternal sacrifice and filial duty—continue to shape caregiving roles in Chinese society. Women, particularly daughters and daughters-in-law, were disproportionately represented among primary caregivers in this study. Many described caregiving as a “moral responsibility” rather than a choice, which contributed to heightened psychological stress and limited opportunities for self-care. This gendered expectation placed women at the intersection of emotional obligation and physical overwork, with significant implications for their health and autonomy. Caregiving was often internalized as a moral role by many women, with limited opportunity for negotiation or refusal These cultural scripts, though sustaining care, also deepened emotional fatigue and invisibility.

**Fig. 1:**
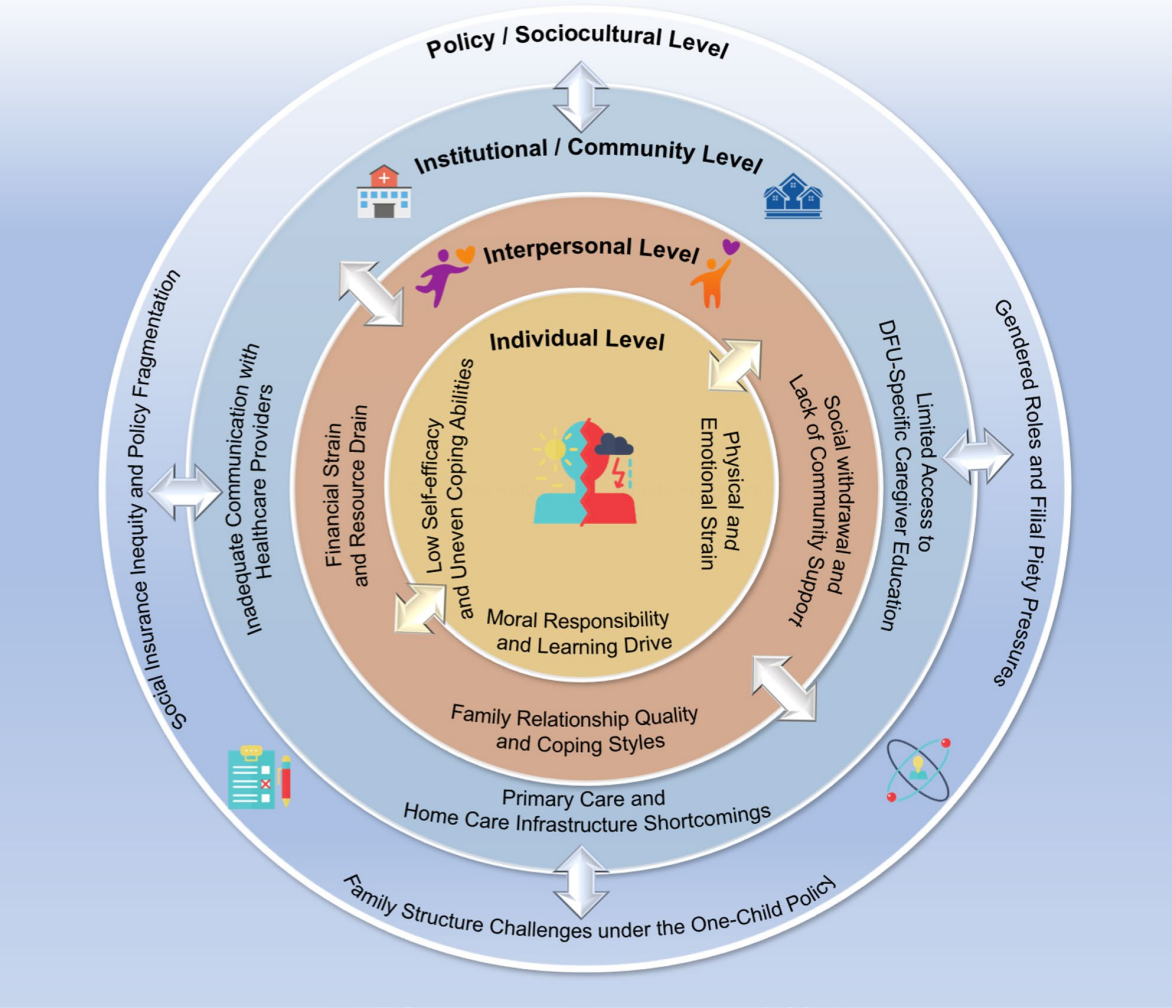
Conceptual framework of family caregiver burden in the care of patients with DFUs based on the Social Ecological Model

## Discussion

### Multilevel drivers of caregiving burden in dfu under the social ecological model

Using the Social Ecological Model (SEM) as an analytic framework, we interpret the caregiving burden and emotional experiences of family caregivers of patients with DFUs as arising from multiple interacting levels, spanning the individual, interpersonal, community, institutional, and sociocultural/policy domains. At the individual level, caregivers experienced profound physical exhaustion, emotional distress, and fluctuating self-efficacy. At the interpersonal level, family support, role sharing, and relationship quality critically shaped caregiving strain. At the community and institutional levels, limited access to structured education, inadequate continuity of care, and fragmented healthcare communication undermined caregivers’ confidence in home-based management. At the sociocultural and policy level, deeply rooted filial norms, gendered caregiving expectations, demographic transitions, and gaps in social welfare further intensified caregiving burden. Together, these multilevel influences highlight that caregiver stress in DFU is not solely an individual issue but the cumulative result of interlocking personal, relational, organizational, and systemic forces. Accordingly, effective interventions must be strategically targeted across multiple SEM levels rather than confined to individual-level support alone. As illustrated in Fig. 1, caregiver burden emerges from the dynamic interaction of multilevel influences across the SEM, while the “upstream–downstream” perspective provides a conceptual lens to interpret how these influences connect across levels.

From a public health perspective, the multilevel patterns observed in this study can be interpreted through an “upstream–downstream” lens, whereby caregiving burden is framed not only as an individual or family concern but as part of a broader population-level challenge in the organization of care and support systems [34]. Within this framework, sociocultural and policy-level influences—such as welfare arrangements, gendered caregiving norms, and demographic structures—shape the availability and organization of family, community, and institutional resources, which in turn condition caregivers’ emotional responses, coping strategies, and everyday care practices [35].

In this sense, caregiver burden in DFU may be understood as emerging from interconnected streams of influence that extend across structural, relational, and individual levels, rather than as the accumulation of independent risk factors.

### Individual-level drivers of caregiving burden: exhaustion and resilience

At the individual level, caregiver burden is primarily driven by physical exhaustion and emotional fatigue, while meaning-based resilience and active learning buffer psychological distress. Physical frailty and emotional fatigue were prominent contributors to the burden of long-term DFU care, with caregivers frequently reporting reduced stamina, emotional strain, and low confidence in managing the fluctuating course of the wound, recurrent infection, and the ongoing risk of ulcer deterioration. These findings are consistent with prior studies linking symptom management challenges to both psychological stress and physical decline [36, 37]. At the same time, caregivers’ psychological resources—such as moral motivation, adaptive learning, and self-directed knowledge seeking—played an important role in resilience. Those who actively sought wound care knowledge and reframed caregiving as a meaningful responsibility demonstrated greater confidence and emotional stability [38, 39]. This suggests that resilience is not an innate trait but rather a dynamic capacity shaped by values, motivation, and learning orientation.

In participants’ accounts, caregivers’ moral motivation was less an individual disposition than an internalization of family and cultural expectations, often articulated through the identity of being a “responsible son” or “responsible daughter”. This role-based moral framing not only shaped caregivers’ commitment but also motivated learning-oriented coping and skill acquisition [21, 22]. Importantly, this internalized responsibility did not yield uniform psychological effects. When embraced as meaningful familial duty, it fostered resilience, persistence, and emotional stability; when experienced as socially imposed and non-negotiable, it was accompanied by guilt, pressure, and role entrapment. This dual pathway suggests that culturally rooted moral motivation may function either as a coping resource or as an added source of strain, depending on caregivers’ subjective interpretation [22].These experiences were further shaped by relational and structural contexts that influenced how responsibility was distributed and sustained [26]. In cases where siblings assumed primary caregiving roles, moral responsibility was not reduced but redistributed, with self-efficacy reconstructed through the enactment of an alternative caregiving position [13, 40]. This illustrates that the designation of the “responsible caregiver” reflects both moral expectation and family structural contingencies.

From this perspective, enhancing caregivers’ psychological resources and caregiving competence becomes central to alleviating individual-level burden. Structured DFU education, targeted skills training, and accessible psychological support may not only strengthen caregivers’ self-efficacy but also help stabilize emotional responses to the uncertainties of long-term wound management, thereby improving the sustainability of home-based DFU care [29, 39].

### Interpersonal drivers of caregiving burden: family support, financial strain, and role imbalance

At the interpersonal level, family support functions as a core protective factor against caregiver burden, whereas role imbalance and financial toxicity substantially amplify caregiving strain. Supportive family environments with shared decision-making and emotional reciprocity were associated with lighter caregiving burden, consistent with family resilience theory [41]. In contrast, caregivers in single-child households or those lacking intra-family support experienced greater overload, underscoring how limited family resources exacerbate vulnerability. These relational dynamics were closely intertwined with financial pressures, as caregivers bore not only direct treatment-related costs—including medications, transportation, specialized dressings, and offloading footwear—but also indirect losses due to reduced employment and declining health. When financial strain constrains access to regular debridement and follow-up visits, family-level burden may further translate into suboptimal treatment adherence and heightened infection risk. Fragmented insurance schemes and restrictive reimbursement policies further intensified these pressures, often forcing families to prioritize coverage limits over clinical needs. Such findings are consistent with previous research identifying financial toxicity as a major contributor to caregiver burden in chronic illness [12, 13].

These dual pressures at the interpersonal level are closely intertwined with systemic constraints, and therefore require integrated interventions across both household and institutional contexts. Early caregiver needs assessments and targeted financial subsidies may help address immediate strains, while broader reforms—such as tiered respite services, integration of public-private support mechanisms, and cost-effective preventive strategies including routine screening and foot care education—could provide more sustainable relief [42–44]. By simultaneously strengthening family caregiving capacity and reducing economic barriers, such cross-level strategies hold promise for improving caregiver well-being and sustaining home-based DFU care.

### Institutional drivers of home-based DFU caregiving burden: education and care continuity

At the community and institutional levels, gaps in caregiver education, provider communication, and continuity of care undermine the effectiveness of home-based DFU management. In practice, limited access to DFU-specific education and insufficient caregiver-provider communication are associated with reduced caregiver confidence, delayed wound recognition, and suboptimal complication prevention. Participants emphasized the need for clear, practical, and tailored guidance for both patients and caregivers. Prior studies similarly indicate that personalized educational strategies—such as one-on-one teaching, motivational interviewing, video tutorials, and mobile applications—can significantly enhance caregiver knowledge and self-efficacy, while empathetic and culturally sensitive communication improves adherence and reduces anxiety [4, 45]. At the institutional level, the involvement of specialist nurses and the formal recognition of caregivers as part of the care team may contribute to optimize home-based DFU management [42].

Beyond conventional education, innovative approaches such as telemedicine and peer support were identified as promising community resources. Telemedicine platforms offering remote consultations, structured educational materials, and accessible home care guidelines were valued for improving continuity of care [46]—particularly for ongoing wound assessment, remote monitoring of exudate and infection signs, and guidance on offloading practices and dressing changes. Similarly, peer educators, including former patients or caregivers, provided experience-based guidance that improved trust and knowledge uptake [37, 42], especially in areas such as daily foot inspection, early recognition of infection, and long-term adherence to preventive foot care behaviors. However, these strategies remain constrained by digital literacy gaps, rural-urban disparities in technological access, and challenges in formalizing peer-support networks. Taken together, the impact of community and institutional interventions appears to hinge not simply on the availability of technologies or services, but on how well these are adapted to local contexts to avoid inadvertently widening existing disparities.

### Structural drivers of caregiving burden in China: policy, gender norms, and welfare gaps

At the sociocultural and policy levels, caregiving burden in China is structurally shaped by the One-Child Policy, gendered caregiving norms, and limited formal welfare protection. The One-Child Policy—introduced in 1980 through the national “Open Letter” that restricted most families to having only one child [47, 48]—fundamentally reshaped family structure, leading to markedly smaller households and diminishing the availability of shared caregiving resources [49]. As a result, demographic changes produced by this policy have often left a single adult child with full responsibility for elder care, a challenge intensified by urban migration and geographic separation [13].

Meanwhile, Confucian values emphasizing filial piety, spousal duty, and maternal sacrifice reinforce expectations—particularly for women—to assume caregiving roles regardless of personal cost [40]. Taken together, filial ethics, demographic contraction, and population mobility converge to concentrate caregiving responsibility within a narrow pool of family members, thereby intensifying individual-level strain. Within this sociocultural and policy landscape, caregiving responsibilities are interpreted and internalized at the individual level, shaping how moral motivation and resilience are formed and sustained [21, 39]. However, our findings also suggest that these same cultural and structural conditions give rise to compensatory collective arrangements that redistribute caregiving demands, a pattern also noted in prior research on family-based care sharing in filial contexts [18]. Rather than being borne exclusively by one individual, responsibilities were at times shared across siblings, extended kin, or co-residing family members, reflecting a relational logic of mutual aid embedded within family networks [18, 23]. In this sense, communitarian caregiving did not replace filial norms but functioned as a moderating mechanism through which filial expectations were operationalized and sustained [22]. This form of shared caregiving reflects a broader communitarian ethos embedded within collectivist family traditions.

When such collective arrangements were further supported through formal mechanisms—such as community-based respite services, peer support initiatives, and nurse-led outreach—caregiving expectations shifted from an exclusively family-centered duty toward a more distributed model of care [18]. These hybrid arrangements illustrate how communitarian values, when reinforced by institutional support, may counterbalance the psychological strain associated with individualized filial responsibility by diffusing role intensity and reducing moral isolation.

In contrast, high-income Western societies rely more heavily on institutionalized mechanisms such as paid caregiving leave, respite services, and long-term care insurance, which can reduce caregiver stress and enhance flexibility [44]. However, these measures may weaken intergenerational ties and undervalue unpaid caregiving work. Compared with China—where cultural norms partially compensate for limited formal support—the Western model reflects a different balance between family and state responsibility. Taken together, caregiving burden in China reflects not only the intensifying effects of filial obligation under demographic and policy constraints, but also the buffering potential of communitarian support systems that redistribute care across relational and institutional networks [22].

Beyond China, similar sociocultural and policy constraints are evident in other non-Western contexts—such as Vietnam, South Korea, and parts of Southeast Asia—where filial obligations and gendered caregiving norms persist amid limited welfare provision [29, 43]. In these settings, caregiving burden reflects not only personal strain but also broader structural and cultural inequities. Rather than adopting Western models wholesale, more feasible strategies include culturally tailored caregiver education, community-based respite care leveraging extended family or faith-based networks, and primary care–home care integration supported by culturally competent health workers. These approaches offer context-sensitive pathways for strengthening caregiver capacity while gradually reducing reliance on informal family labor.

### Implications for nursing practice

The multilevel drivers identified in this study suggest that translating these findings into nursing practice requires support strategies that are responsive to caregivers’ points of uncertainty, overload, and declining confidence, rather than delivered as a uniform or solely patient-focused intervention.

At the individual level, caregivers in multiple interviews described learning wound care through trial and error, often accompanied by anxiety about making mistakes or missing early signs of deterioration. Nurses can contribute by providing structured, task-focused guidance on wound assessment, dressing techniques, and infection recognition, alongside brief, supportive communication that normalizes uncertainty and encourages questions during follow-up. Such approaches can strengthen caregiver confidence and engagement in home-based DFU management [4, 45].

At the family level, participants’ accounts reflected uneven role distribution and strain related to balancing care responsibilities, employment, and financial pressures. Incorporating additional family members into care discussions—particularly during outpatient consultations or discharge planning—may help clarify caregiving roles and identify informal sources of support before strain escalates into burnout, consistent with recommendations from the family caregiving literature [41].

Beyond the household, gaps in continuity of care and fragmented communication were particularly evident during transitions from hospital to home. Periodic nurse-led follow-up, through telephone or telehealth platforms, can reinforce care instructions and address emerging concerns. Evidence from DFU and other chronic wound contexts suggests that such approaches, when embedded in routine care pathways, have been associated with improvements in care continuity and reductions in potentially avoidable complications [15].

Taken together, these observations suggest the value of embedding nursing support within existing clinical workflows and community services, so that professional input complements—rather than replaces—the family and community-based care arrangements that caregivers already rely on.

## Limitations

This study offers novel insights into the multilevel influences on caregiving burden in the context of diabetic foot ulcers, drawing on the Social Ecological Model and rich qualitative data from semi-structured interviews. However, several limitations should be acknowledged.

First, the sample was relatively small and drawn exclusively from Shanghai, which may limit the transferability of the findings to other regions of China with different healthcare resources, socioeconomic conditions, and cultural contexts. In particular, caregiving experiences in rural and less-developed areas were not sufficiently represented. In addition, the inclusion and exclusion criteria may have introduced selection bias, as caregivers with severe physical conditions or formally diagnosed psychiatric disorders were not included. As a result, the findings may not fully capture the experiences of more vulnerable caregivers who could experience higher levels of burden and psychological strain. Future urban-rural comparative studies may clarify how differences in healthcare access and social support shape caregiving burden, while longitudinal designs could capture changes in caregiving experiences and support needs over time.

Second, as the data were based on self-reported interviews, the findings may have been affected by social desirability bias. This is particularly relevant in the Chinese sociocultural context, where strong filial piety norms emphasizing moral duty, endurance, and self-sacrifice may lead caregivers to underreport emotional distress, exhaustion, or burden, potentially resulting in an underestimation of the full extent and complexity of caregiving burden.

In addition, although integrating Colaizzi’s phenomenological analysis with the Social Ecological Model provided a theoretically structured interpretation, the process of situating inductively derived themes across the SEM levels may have involved a degree of analytical simplification. Some caregiving experiences were inherently cross-level, spanning individual, interpersonal, and sociocultural or policy domains, and the boundaries between participants’ narratives and our SEM-informed interpretation may therefore not be entirely discrete. This should be taken into account when interpreting the multilevel structure of the findings. Future studies could enrich understanding of caregiver burden through triangulation using observational data, longitudinal follow-up, or multiple family perspectives.

## Conclusions

This study revealed the multilevel burden faced by family caregivers of patients with DFUs in China, shaped by individual, familial, institutional, and cultural factors. Guided by the Social Ecological Model, the findings highlight the need for system-level, culturally sensitive interventions such as caregiver education, respite services, and long-term care policy reforms. While rooted in the Chinese context, these insights are transferable to other non-Western societies where filial obligations and constrained welfare systems similarly shape caregiving. By linking culture, policy, and caregiving practices, this study provides a foundation for developing context-specific strategies to support chronic disease care in resource-limited settings.

## Electronic Supplementary Material

Below is the link to the electronic supplementary material.

Supplementary Table S1 Consolidated Criteria for Reporting Qualitative Research (COREQ) Checklist and Reporting Locations

## Data Availability

The data that support the findings of this study are available on request from the corresponding author. The data are not publicly available due to privacy or ethical restrictions.
