# Supplementary material for: Multilevel influences on family caregiver burden in diabetic foot ulcer care: a qualitative study
Source: BMC Nurs. 2026 Mar 30;25:555. doi: 10.1186/s12912-026-04577-0 (PMC13289486; doi:10.1186/s12912-026-04577-0)
Supplement: Supplementary file 1 — Supplementary Table S1 Consolidated Criteria for Reporting Qualitative Research (COREQ) Checklist and Reporting Locations [file 12912_2026_4577_MOESM1_ESM.docx]

**Supplementary Table S1 Consolidated Criteria for Reporting Qualitative Research (COREQ) Checklist and Reporting Locations**

**Table S1. Combined criteria for reporting qualitative research (COREQ)**

| Item | Guide Questions/Description | Reported in Manuscript |
| --- | --- | --- |
| **Domain 1: Research team and reflexivity** | |  |
| 1. Interviewer/facilitator | Which author/s conducted the interview or focus group? | YL and ZG conducted the interviews. |
| 2. Credentials | What were the researcher's credentials? | Researchers had nursing and qualitative research backgrounds. |
| 3. Occupation | What was their occupation at the time of the study? | Nurse researchers and a senior wound care nurse.. |
| 4. Gender | Was the researcher male or female? | One male (ZG) and one female (YL) conducted the interviews. |
| 5. Experience and training | What experience or training did the researcher have? | Trained in qualitative interviewing and wound care practice. |
| 6. Relationship established | Was a relationship established prior to study commencement? | No prior relationship was established. |
| 7. Participant knowledge | What did the participants know about the researcher? | Participants were informed that the interviewers were independent academic researchers with no role in their clinical care and that the study aimed to explore family caregiving experiences for research purposes. |
| 8. Interviewer characteristics | What characteristics were reported about the interviewer/facilitator? | Researchers acknowledged their clinical background and minimized cultural bias through reflexive discussions. |
| **Domain 2: Study design** | |  |
| 9. Methodological orientation | What methodological orientation was stated to underpin the study? | Descriptive qualitative design informed by SEM and phenomenological analysis |
| 10. Sampling | How were participants selected? | Purposive sampling of family caregivers of patients with DFU. |
| 11. Method of approach | How were participants approached? | Face-to-face recruitment in hospital settings. |
| 12. Sample size | How many participants were in the study? | 16 family caregivers. |
| 13. Non-participation | How many people refused to participate or dropped out? | No participants refused or withdrew; all 16 completed interviews. |
| 14. Setting of data collection | Where was the data collected? | Hospital consultation rooms and medical offices. |
| 15. Presence of non-participants | Was anyone else present besides the participants and researchers? | Only participants and interviewers were present. |
| 16. Description of sample | What are the important characteristics of the sample? | Demographic and caregiving-related information provided in **Tables 3.** |
| 17. Interview guide | Were questions, prompts, guides provided by the authors? | Yes; Developed from systematic reviews, mapped to SEM levels, reviewed by clinical experts, and pilot tested with two caregivers. |
| 18. Repeat interviews | Were repeat interviews carried out? | No repeat interviews were conducted. |
| 19. Audio/visual recording | Did the research use audio or visual recording to collect the data? | Audio-recorded interviews. |
| 20. Field notes | Were field notes made during and/or after the interview or focus group? | No formal field notes were reported. |
| 21. Duration | What was the duration of the interviews or focus group? | Approximately 30 minutes per interview. |
| 22. Data saturation | Was data saturation discussed? | Yes, data saturation was reached. |
| 23. Transcripts returned | Were transcripts returned to participants for comment and/or correction? | Transcripts were not returned to participants; instead, a verbal summary of each interview was provided at the end of the interview for confirmation of accuracy. |
| **Domain 3: Analysis and findings** | |  |
| 24. Number of data coders | How many data coders coded the data? | Two researchers independently coded the data. |
| 25. Description of the coding tree | Did authors provide a description of the coding tree? | Themes and sub-themes have been presented in the Results section and corresponding tables. |
| 26. Derivation of themes | Were themes identified in advance or derived from the data? | Themes were derived using a combination of inductive and deductive approaches. |
| 27. Software | What software, if applicable, was used to manage the data? | NVivo 12 Plus was used to manage and organise qualitative data; all coding and theme development were conducted by researchers. |
| 28. Participant checking | Did participants provide feedback on the findings? | Participants did not formally review or provide feedback on the derived themes or findings; instead, analytic credibility was supported through repeated transcript checks, independent double coding, team-based consensus discussions, and bilingual review of translated quotations. |
| 29. Quotations presented | Were participant quotations presented to illustrate the themes/findings? | Yes, quotations are presented in **Table 4.** |
| 30. Data and findings consistent | Was there consistency between the data presented and the findings? | Yes, consistency is demonstrated between data and themes. |
| 31. Clarity of major themes | Were major themes clearly presented in the findings? | Yes, four major themes are clearly presented. |
| 32. Clarity of minor themes | Is there a description of diverse cases or minor themes? | Yes, subthemes and variations are discussed within each theme. |
